# Exploring the Interaction of *Drosophila* TDP-43 and the Type II Voltage-Gated Calcium Channel, *Cacophony*, in Regulating Motor Function and Behavior

**DOI:** 10.1177/1179069517740892

**Published:** 2017-11-15

**Authors:** Kayly M Lembke, David B Morton

**Affiliations:** 1 Department of Physiology & Pharmacology, Oregon Health & Science University, Portland, OR, USA; 2 Department of Integrative Biosciences, Oregon Health & Science University, Portland, OR, USA

**Keywords:** ALS, amyotrophic lateral sclerosis, *Drosophila melanogaster*, neurodegeneration, voltage-gated ion channel

## Abstract

Amyotrophic lateral sclerosis (ALS) is the most common adult onset motor neurodegenerative disease. The cause of the disease remains obscure, and as such there is no effective treatment or cure. Amyotrophic lateral sclerosis and other neurodegenerative diseases are frequently characterized by dysfunction of the RNA-binding protein, TDP-43. Using model systems to understand the mechanisms underlying TDP-43 dysfunction should accelerate identification of therapeutic targets. A recent report has shown that motor defects caused by the deletion of the *Drosophila* TDP-43 ortholog, *tbph*, are not driven by changes in the physiology at the neuromuscular junction. Rather, defective motor burst rhythmicity and coordination, displayed by *tbph* mutants, are rescued by genetically restoring a voltage-gated calcium channel to either motor neurons or just a single pair of neurons in the brain. If these effects are mirrored in human TDP-43 proteinopathies, these observations could open new avenues to investigate alternative therapeutic targets for these neurodegenerative diseases.

Amyotrophic lateral sclerosis (ALS) is the most common adult onset motor neurodegenerative disease that has been characterized.^1^ Although it was first described as a distinct disease in 1864, the mechanisms of disease progression remain poorly understood. Part of the reason for this lack of understanding is that most of our understanding of the cellular and molecular basis of the disease comes from postmortem patient tissue samples, providing little information on the early defects associated with motor neuron degeneration. Approximately 96% of ALS patient tissues show a mislocalization of the nuclear protein TDP-43 from the nucleus, to the cytoplasm of motor neurons, where it forms insoluble protein aggregates.^2^ TDP-43 proteinopathy is also observed in a significant number of other neurodegenerative diseases, including Alzheimer disease and frontotemporal dementia.^3^ There is, however, evidence that the formation of these aggregates themselves is not sufficient to confer cytotoxicity.^4,5^ Therefore, understanding the downstream effects of the loss of nuclear TDP-43 function could explain the development and progression of motor failure in patients with ALS, as well as identify potential therapeutic targets in ALS and other neurodegenerative diseases.

To model the loss of nuclear function, we have used the power of *Drosophila* genetics to remove the endogenous *Drosophila* TDP-43 ortholog, named *tbph*,^6^ and investigate the effects of interacting genes. TBPH is a nuclear, RNA-binding protein that regulates the expression of more than 1000 different gene transcripts,^6^ including the gene *cacophony*, which encodes the type II voltage-gated calcium channel necessary for full evoked neurotransmission at the *Drosophila* neuromuscular junction (NMJ).^7^ Loss of *tbph* causes a significant reduction in the expression of *cacophony* protein.^6,8^ We and others have shown that loss of *tbph* also causes severe motor defects resulting in a reduction in the total distance crawled by third instar *Drosophila* larvae.^6,8,9^ In addition to shorter distances crawled, these third instar larvae show severely defective peristaltic wave progression and frequency.^10^ Using the GAL4-UAS system, we were able to test whether these motor defects could be rescued by genetically restoring *cacophony* to all neurons, specifically motor neurons, and subsets of neurons known to modulate motor behavior. Surprisingly, despite a large number of genes being affected by loss of *tbph*, restoration of the levels of only a single gene, *cacophony*, was sufficient to restore the defects in larval locomotion.^8,10^

Evidence accumulated across model systems has traditionally suggested that ALS is a purely motor neuron disease, and much emphasis has been placed on understanding the effects of TDP-43 loss-of-function and gain-of-function in motor neurons. Recently, it was revealed, in a transgenic inducible rNLS8 mouse model that forms both cytoplasmic TDP-43 proteinopathy and loss of TDP-43 nuclear localization, that 7 motor neuron pools were found to respond differently to TDP-43 proteinopathy.^11^ Motor neurons in the oculomotor, trigeminal, and facial nuclei were unaffected.^12^ Motor neurons in the hypoglossal nucleus and the spinal cord were lost after 8 weeks of transgenic expression.^12^ Therefore, it was not surprising when, using the motor neuron specific drivers D42-GAL4 and OK6-GAL4, genetically restoring *cacophony* in all motor neurons in the larval central nervous system (CNS) rescued the severe crawling defects of the *tbph* mutants.^8,10^

Smaller subsets of neurons in the larval brain have been shown to modulate crawling behavior.^13^ Because different pools of motor neurons in vertebrate systems are sensitive to changes in TDP-43 expression, we tested whether genetically restoring *cacophony* in subsets of neurons known to modulate motor behavior would be sufficient to rescue the motor defects of the TBPH mutant. Using a variety of GAL4 drivers specific to subsets of motor neurons and neurons that act on the motor circuit,^13^ we found that driving *cacophony* in a single pair of cells in the larval brain was sufficient to rescue larval crawling and peristaltic wave progression to the level achieved using the D42-GAL4 motor neuron driver.

The pair of cells, called the AVM001b/2b cells, are housed bilaterally in the protocerebrum of the larval brain hemispheres.^10,13^ They do not project out of the brain to directly innervate the body wall musculature and do not appear to directly interact with the motor neurons housed in the neuromeres of the ventral nerve cord, the equivalent of the spinal cord. Their activation by channel rhodopsin has been shown to drive mild escape behavior,^13^ but beyond this recorded observation, nothing is known about them functionally. Nonetheless, we have shown that genetically restoring *cacophony* in this single pair of cells in the brain, which do not form a NMJ at body wall muscles, is sufficient to restore motor defects associated with loss of TBPH. We believe that this finding has important implications to understanding the mechanisms underlying the phenotypic effects of TDP-43 dysfunction.

We sought to understand the mechanism of this *cacophony*-specific rescue. The NMJ is the structure at which innervation of muscles by motor neurons occurs. As *cacophony* is the primary voltage-gated calcium channel at this structure, evoked neurotransmission at the NMJ is sensitive to changes in *cacophony* channel expression.^7,14,15^ We therefore expected that motor defects presented by *tbph* mutants would be driven by changes to the physiology of neurotransmission at the NMJ. It was surprising, therefore, that this was not the case; *tbph* mutants showed no defects in evoked neurotransmission, but did exhibit defects in spontaneous neurotransmitter release. We demonstrated a *tbph*-dependent decrease in miniature end-plate potential amplitude, which was not *cacophony* dependent, as well as a reduction in the frequency of spontaneous release, which was *cacophony* dependent.^10^ These defects, however, were not sufficient to explain the decrease in crawling distance and defects to peristaltic wave progression and frequency.^10^ Therefore, it appeared that the *cacophony*-dependent motor defect, present in the *tbph* mutants, was not due to changes in the physiology at the NMJ.

Classically, motor neurons were thought to be passive followers of the patterned motor output of central pattern generators in the CNS.^16^ More recently, however, it has been reported that motor neurons, themselves, act to refine and regulate the progression of the peristaltic wave; silencing of motor neurons in a single neuromere was sufficient to halt the progression of the peristaltic wave.^17^ We therefore tested whether *tbph* mutants showed an altered motor pattern produced from a semi-isolated CNS. To do this, extracellular recordings performed on intact motor nerves of the *tbph* mutant were made. These recordings revealed unpatterned, unrhythmic motor bursts that lacked coordination between segmental bursts.^10^ Restoring *cacophony* in all motor neurons, using the D42-GAL4 driver, restored motor rhythmicity and coordination.^10^ But the question was glaring: Was this rescue dependent upon restoring *cacophony* solely in motor neurons? Genetically restoring *cacophony* in the AVM001b/2b cells in the *tbph* mutant background also restored rhythmic motor bursts, motor burst coordination, peristaltic wave progression, and distance crawled.^10^ This result was unexpected, and it suggests that broad, systemic motor defects can be driven by the loss of a single gene in a pair of neurons that do not directly innervate body wall musculature.

Beyond their function in driving escape behavior, what function the AVM001b/2b cells may play endogenously in the motor circuit, and what neurotransmitters these cells release and receptors they express, is still unclear. As restoring *cacophony* in the AVM001b/2b cells recapitulates the rescue achieved by restoring *cacophony* in all motor neurons, it would appear that the AVM001b/2b cells act upstream of the motor neurons housed in the neuromeres. However, simply activating the AVM001b/2b cells in the *tbph* mutant background failed to restore larval locomotion and killing them crudely, using UAS-*rpr*, had no effect on larval crawling or development, suggesting instead they do not directly act to modulate the motor circuit.^10^ Rather, the AVM001b/2b cells could be part of a divergent pathway that is sufficient to modulate motor behavior but not necessary for normal motor function.

In addition to the unknown function of these cells, it is also unknown whether these cells endogenously express *cacophony* and, if so, which isoforms of *cacophony* they express. There are 14 reported isoforms of *cacophony* (http://flybase.org/). Voltage-gated calcium channels assume several functions within the cell, which include mediating inward calcium currents that depolarize the cellular membrane potentials, mediating excitability, and providing intracellular calcium signals that can activate gene transcription and neurotransmitter release.^18–21^ One mechanism cells can use to achieve functional specificity is in subtle changes in channel isoform expression caused by differential splicing.^21,22^

TBPH functions to regulate transcript splicing, and Chang et al^8^ showed that *tbph* mutants express an enrichment of *cacophony* transcripts lacking a specific exon, exon 7, which codes for a region located near the C-terminus of the protein. This observation was of interest because of the 14 reported *cacophony* transcripts, only 1 lacks exon 7 (http://flybase.org/), suggesting that exon 7 confers some critical function. Analyses on the population of voltage-gated calcium channel isoforms in different mouse brain regions, throughout development, has shown that the composition of voltage-gated calcium channel messenger RNA splice isoforms varies by cell type, development stage, and neuronal activity.^19^ Therefore, enrichment in the expression of *cacophony* transcripts lacking exon 7 could potentially contribute to the *cacophony*-dependent motor defects of *tbph* mutants. Although we cannot speak for certain on whether the lack of exon 7 is sufficient to drive whole animal motor defects, tools such as CRISPR/cas9 will enable these questions to be explored.

TDP-43 proteinopathies are characteristic of several neurodegenerative diseases whose causes remain obscure. Understanding the downstream effects of the loss of TDP-43 in discrete subsets of cells, that do not directly innervate musculature, forces a shift in paradigm surrounding the subsequent motor defects. Furthermore, not only have we shown that it is the loss of the TDP-43 in a discrete pair of cells that drives the motor defect, but the resulting loss of a single gene, *cacophony*, that directly causes them. These observations are novel and, although they result in more questions than they solve, they ultimately shed light on the sensitivity of the motor circuit to the downstream effects from changes in TDP-43 expression. They suggest that it is a restricted loss of the TDP-43, and not a whole-systems loss, which drives motor defects.
